# Development and Validation of a Protein Electrophoresis Classification Algorithm: Tabular Data-Based Alternative

**DOI:** 10.2196/83124

**Published:** 2026-01-28

**Authors:** Auriane Mazuir, Gatien Ricotier, Pierre Filhine-Tresarrieu

**Affiliations:** 1 Laboratoire B2A Brumath France; 2 Institut de Recherche Mathématique Avancée Strasbourg France

**Keywords:** machine learning, CatBoost, serum protein electrophoresis, convolutional neural network, tabular data analysis, clinical informatics, diagnostic interpretation, computational efficiency

## Abstract

Serum protein electrophoresis (SPE) is routinely interpreted through visual assessment of electropherogram images by medical laboratory scientists. We introduce an efficient tabular data–based machine learning approach that directly leverages numerical SPE profiles, offering a robust and interpretable alternative to image-based deep learning methods.

## Introduction

Serum protein electrophoresis (SPE) is a key technique for separating and quantifying major serum protein fractions. Recent studies [1-3] have used convolutional neural networks (CNNs) to classify SPE results. Although these models have shown good performance, they primarily replicate the visual interpretation performed by medical laboratory scientists (MLS). Yet electropherograms are inherently numerical curves—that is, tabular data. This raises a simple question: why analyze an image when the analytical signal already exists as a numerical table?

Although image-based CNNs remain the dominant approach, we explicitly reframe SPE classification as a purely tabular learning problem concerning numerical SPE profiles. We evaluate this perspective by comparing our approach to the CNN-based study of Lee et al [1] by using the same dataset [4] and identical training-test splits, without additional data cleaning or hyperparameter tuning.

## Methods

**Figure 1:**
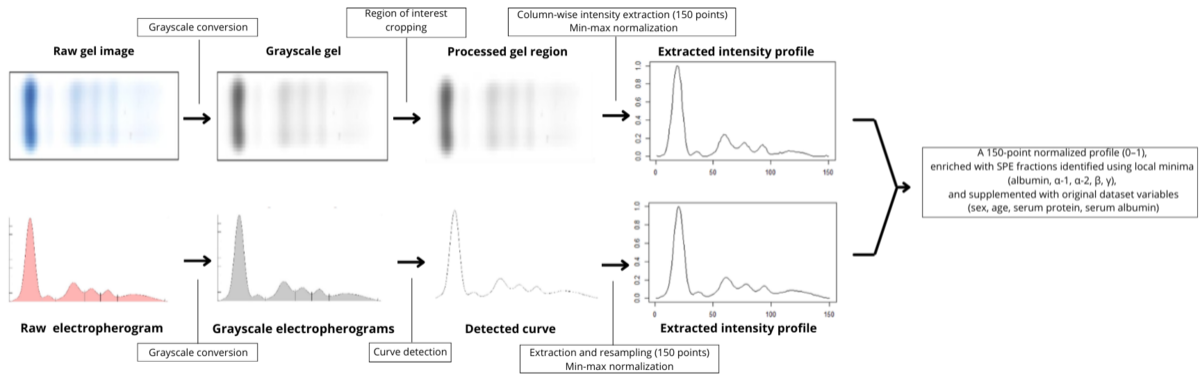
Preprocessing steps showing the tabular data extraction for both types of serum protein electrophoresis images.

Input data were obtained by extracting numerical profiles from electropherograms and gel images as illustrated in Figure 1. Each image underwent grayscale conversion, cropping of the analytical region, interpolation into 150 point profiles, and min-max normalization. We computed SPE fractions by using local-minima detection (albumin, α-1, α-2, β, γ) and included demographic and biochemical variables from the dataset (sex, age, serum protein, serum albumin).

The 6 pathological categories defined in the reference dataset [4] were acute phase protein increase (74 cases), monoclonal gammopathy (264 cases), polyclonal gammopathy (244 cases), hypoproteinemia (249 cases), nephrotic syndrome (165 cases), and normal profiles (293 cases). Each case corresponds to a specific distribution pattern of proteins. These SPEs were collected in [1] between January 2018 and July 2019.

As recently emphasized [5,6], tree-based gradient boosting models remain the strongest performers for tabular data, often surpassing deep learning. After converting SPE images into numerical matrices, we reconfirmed this by evaluating XGBoost (extreme gradient boosting), TabPFN (tabular foundation model), and CatBoost (categorical boosting). Without any hyperparameter optimization, CatBoost consistently produced the best results, especially on the gel-extracted data.

All results were obtained with the default CatBoost parameters from R implementation [7] running on R software (version 4.4.3; R Foundation for Statistical Computing) [8]. To enable a fair comparison to the CNN baseline in [1], we used the exact same training and test splits as in [1]: specifically, 10% of the cases were reserved for testing. However, the distribution of these cases differed between gel and electropherogram representations. Each experiment was repeated with 100 different seeds to estimate CIs for all performance metrics.

## Results

In total, 1289 SPE cases were available, each providing a gel and an electropherogram. However, the image quality varied substantially across samples: gel image heights ranged from 29 to 556 pixels (mean 81.9, SD 48.1) and widths from 96 to 876 pixels (mean 275, SD 129); electropherogram images heights ranged from 98 to 704 pixels (mean 410.7, SD 183.1) and widths from 250 to 1075 pixels (mean 649.6, SD 288.3) (Table 1).

Among the 20 polyclonal gammopathy cases in the gel test set, the sensitivity reported by [1] is 0.800, whereas our approach achieves a mean sensitivity of 0.941, with a 95% CI of 0.937-0.945 across 100 repeated runs. Except for monoclonal gammopathies where we, by contrast with [1], removed the unusual spike delimitation in our preprocessing step, the CatBoost-based tabular approach outperformed the CNN baseline across most categories. Weighted sensitivity, specificity, and *F*_1_-scores were improved when using tabular data rather than images.

**Table 1:** Sensitivities, specificities, and F1-scores for protein electropherograms and gels, comparing the original model with the average performance of our model over 100 repeated runs, stratified by pathology, with weighted averages computed over the entire dataset.

	Sens^a^ Ref^b^ Electro^c^	Sens CatBoost^d^ Electro	Spec^e^ Ref Electro	Spec CatBoost Electro	*F*_1_-score Ref Electro	*F*_1_-score CatBoost Electro	Sens Ref Gel	Sens CatBoost Gel	Spec Ref Gel	Spec CatBoost Gel	*F*_1_-score Ref Gel	*F*_1_-score CatBoost Gel
Acute phase protein (n^f^=5 or 9)	*0.600* ^g^	0.594 (0.587-0.601)	0.951	*0.990* (0.990-0.991)	0.429	*0.648* (0.640-0.655)	0.222	*0.441* (0.437-0.445)	0.882	*0.993* (0.992-0.994)	0.160	*0.576* (0.571-0.582)
Monoclonal gammopathy (n=29 or 24)	*0.862* ^h^	0.690 (0.689-0.691)	*1.000* ^h^	0.984 (0.983-0.986)	*0.926* ^h^	0.792 (0.790-0.794)	*0.792*	0.658 (0.650-0.665)	0.981	*0.998* (0.997-0.999)	*0.844*	0.788 (0.783-0.793)
Polyclonal gammopathy (n=22 or 20)	0.818	*1.000* (1.000-1.000)	*0.981*	0.978 (0.977-0.979)	0.857	*0.950* (0.947-0.953)	0.800	*0.941* (0.937-0.945)	0.917	*0.983* (0.982-0.984)	0.711	*0.925* (0.923-0.928)
Hypoproteinemia (n=26 or 25)	0.846	*0.878* (0.874-0.881)	0.853	*0.974* (0.973-0.975)	0.698	*0.887* (0.884-0.889)	0.520	*0.831* (0.827-0.834)	0.893	*0.898* (0.896-0.899)	0.531	*0.738* (0.735-0.740)
Nephrotic syndrome (n=16 or 21)	0.687	*0.853* (0.845-0.861)	*0.991*	0.0.954 (0.953-0.955)	*0.786*	0.783 (0.778-0.788)	0.238	*0.699* (0.692-0.706)	*0.972*	0.944 (0.942-0.945)	0.345	*0.704* (0.698-0.709)
Normal (n=30 or 29)	0.667	*0.920* (0.914-0.925)	*0.949*	0.939 (0.939-0.939)	0.727	*0.868* (0.865-0.870)	0.759	*0.935* (0.932-0.937)	0.879	*0.925* (0.923-0.927)	0.698	*0.854* (0.851-0.856)
Weighted scores	0.773	*0.852* (0.850-0.854)	0.952	*0.967* (0.966-0.967)	0.784	*0.849* (0.848-0.851)	0.602	*0.790* (0.788-0.792)	0.922	*0.950* (0.950-0.951)	0.599	*0.786* (0.784-0.788)

^a^Sens: sensitivity.

^b^Ref: reference.

^c^Electro: electropherogram.

^d^CatBoost: categorical boosting.

^e^Spec: specificity.

^f^The values of n correspond to the number of test samples for electropherograms and gels, respectively. Values in parentheses report the 95% CIs of our model.

^g^Italicized values indicate the best-performing model for each metric and category.

^h^Denotes the use of unusual spike delimitation on all electropherograms of monoclonal gammopathies.

## Discussion

The main limitation appears in the monoclonal gammopathy class [9] on electropherograms, for which CNNs in [1] report higher performance. A plausible explanation lies in the structure of the original dataset: in [4], electropherograms corresponding to monoclonal gammopathies systematically contain manually drawn spike delimitations added by MLS during routine interpretation. These annotations are specific to this class and may therefore serve as highly discriminative visual cues for the convolutional model, artificially boosting its performance. In contrast, this dataset bias is removed from our tabular dataset using our preprocessing pipeline. It removes all such manual markings to retain a purely signal-based representation, thereby eliminating visual hints that CNN may have leveraged in the original setting. Despite this bias, our framework achieves stable and homogeneous performance across all pathological categories and does not show a specific degradation for monoclonal gammopathies.

Our CatBoost results were obtained using the default parameters, without any form of tuning. This choice was intentional: it demonstrates that even an entirely nonoptimized tabular model already outperforms the CNN baseline on most categories, even on a dataset with several low quality images. Consequently, additional improvements are highly plausible. More extensive hyperparameter optimization such as tuning tree depth, learning rate, and boosting iterations could further enhance performance. Likewise, hybrid approaches that enrich numerical profiles with peak-shape descriptors or selectively integrate localized image-based features may help address the specific challenges posed by narrow M-spikes in monoclonal gammopathies.

Reframing SPE classification as a tabular learning task leads to immediate performance improvements, even before any optimization. Beyond accuracy, this approach offers several practical advantages. First, the approach is computationally efficient: CatBoost trains rapidly on a standard laptop and requires no graphics processing unit, and it integrates easily into routine workflows. Second, this approach is readily generalizable, especially with modern SPE analyzers that already store raw numerical curves internally. It means the classification model can be applied directly to these exported values without any image-processing pipeline. Finally, tree-based models offer greater interpretability, allowing laboratories to analyze feature importance and understand which parts of the curve contribute to the classification—a key requirement for clinical use. Moreover, unsurprisingly, this framework is image type agnostic: when exchanging the train-test split for electropherogram and gel, performance remains consistent. This confirms that the improvement comes from the change in data structure rather than from the image source itself.

In summary, transitioning from image-based deep learning to tabular data-based machine learning increases performance and improves robustness, interpretability, reproducibility, and ease of deployment. This redefinition of the SPE classification problem, unconventional for practitioners yet natural for computational systems, provides a compelling alternative to CNN-based approaches and a promising basis for clinically reliable automation.

## Appendix

Multimedia Appendix 1 Use of generative artificial intelligence for code development.

## Abbreviations

CatBoost: categorical boosting
CNN: convolutional neural network
MLS: medical laboratory scientists
SPE: serum protein electrophoresis
TabPFN: tabular foundation model
XGBoost: extreme gradient boosting

## Data Availability

All data used in this study are publicly accessible through the Harvard Dataverse repository, as released by Lee [4].
