# Supplementary material for: Development and Validation of a Protein Electrophoresis Classification Algorithm: Tabular Data-Based Alternative
Source: JMIR Form Res. 2026 Jan 28;10:e83124. doi: 10.2196/83124 (PMC12895147; doi:10.2196/83124)
Supplement: Multimedia Appendix 1 [file formative_v10i1e83124_app1.docx]

**Appendix 1 — Use of Generative AI for Code Development**

Generative artificial intelligence tools were used to assist in the development and refinement of the computational code employed in this study. Specifically, ChatGPT (OpenAI) was used under full human supervision during several non-decisional stages of the coding process.

ChatGPT was used to:

1. draft initial code templates and structural skeletons for data preprocessing and feature extraction pipelines;
2. suggest improvements to code organization, readability, and modularity;
3. assist in identifying and correcting syntax errors and implementation issues during debug- ging;
4. propose alternative implementations for routine operations such as data handling and visu- alization.

ChatGPT was not used to design the study, define the methodological framework, select models, choose evaluation metrics, tune hyperparameters, or interpret experimental results. All algorithmic choices, validation strategies, and analytical decisions were independently made by the authors.

All code generated with the assistance of ChatGPT was systematically reviewed, modified when necessary, and validated by the authors prior to its use in the experiments. The authors take full responsibility for the correctness, originality, and integrity of the final codebase and for all results reported in this manuscript.
